# Pockets of HIV Non-infection Within Highly-Infected Risk Networks in Athens, Greece

**DOI:** 10.3389/fmicb.2018.01825

**Published:** 2018-08-24

**Authors:** Leslie D. Williams, Evangelia-Georgia Kostaki, Eirini Pavlitina, Dimitrios Paraskevis, Angelos Hatzakis, John Schneider, Pavlo Smyrnov, Andria Hadjikou, Georgios K. Nikolopoulos, Mina Psichogiou, Samuel R. Friedman

**Affiliations:** ^1^Institute for Infectious Disease Research, National Development and Research Institutes, New York, NY, United States; ^2^Department of Hygiene, Epidemiology and Medical Statistics, Medical School, National and Kapodistrian University of Athens, Athens, Greece; ^3^Transmission Reduction Intervention Project, Athens, Greece; ^4^Departments of Medicine and Public Health Sciences, University of Chicago Medical Center, Center for AIDS Elimination, Chicago, IL, United States; ^5^Alliance for Public Health, Kyiv, Ukraine; ^6^Medical School, University of Cyprus, Nicosia, Cyprus and European University Cyprus, Nicosia, Cyprus; ^7^Medical School, University of Cyprus, Nicosia, Cyprus; ^8^First Department of Internal Medicine, Laikon General Hospital, Medical School, National and Kapodistrian University of Athens, Athens, Greece

**Keywords:** networks, HIV transmission, non-infection, HIV risk, firewall effects, bottleneck effects

## Abstract

As part of a network study of HIV infection among people who inject drugs (PWID) and their contacts, we discovered a connected subcomponent of 29 uninfected PWID. In the context of a just-declining large epidemic outbreak, this raised a question: What explains the existence of large pockets of uninfected people? Possible explanations include “firewall effects” (Friedman et al., 2000; Dombrowski et al., 2017) wherein the only HIV+ people that the uninfected take risks with have low viral loads; “bottleneck effects” wherein few network paths into the pocket of non-infection exist; low levels of risk behavior; and an impending outbreak. We considered each of these. Participants provided information on their enhanced sexual and injection networks and assisted us in recruiting network members. The largest connected component had 241 members. Data on risk behaviors in the last 6 months were collected at the individual level. Recent infection was determined by LAg (Sedia^TM^ Biosciences Corporation), data on recent seronegative tests, and viral load. HIV RNA was quantified using Artus HI Virus-1 RG RT-PCR (Qiagen). The 29 members of the connected subcomponent of uninfected participants were connected (network distance = 1) to 17 recently-infected and 24 long-term infected participants. Fourteen (48%) of these 29 uninfected were classified as “extremely high risk” because they self-reported syringe sharing and had at least one injection partner with viral load >100,000 copies/mL who also reported syringe sharing. Seventeen of the 29 uninfected were re-interviewed after 6 months, but none had seroconverted. These findings show the power of network research in discovering infection patterns that standard individual-level studies cannot. Theoretical development and exploratory network research studies may be needed to understand these findings and deepen our understanding of how HIV does and does not spread through communities. Finally, the methods developed here provide practical tools to study “bottleneck” and “firewall” network hypotheses in practice.

## Introduction

We present a case study of a large sub-network of *non-infection* that we encountered during the Transmission Reduction Intervention Project (TRIP). TRIP traced the injection and sexual networks of recently-infected people in a successful attempt to recruit and intervene with additional recently-infected people to get them into treatment both to protect their health and to reduce their transmitting HIV to others during the early infection period of high viral load. In the course of this project, we discovered a large, connected sub-component of 29 *uninfected people* within a larger network that contained many recently-infected members.

This paper explores how such a large connected “pocket” of the uninfected could exist. It considers three possible explanations for the existence of such a sub-network:

1. The “bottleneck effect” (Klovdahl, 1985). A network bottleneck can be said to exist if there are very few risk network links between a subnetwork of the uninfected and a subnetwork that contains infected people.
2. The “firewall effect” (Friedman et al., 2000; Dombrowski et al., 2013a,b; Khan et al., 2013). In the firewall effect, long-term infected people with low viral loads who link highly-infectious newly infected people to uninfected people essentially greatly reduce potential transmission between the two groups. In practice, a firewall effect would be observed if the only links uninfected people have with infected people are with those who have low viral loads and have been infected for a year or more.
3. Low levels of risk behavior. Usually, this would be studied by determining if the uninfected participants in a study reported engaging rarely or never in high-risk behavior. In a network study in which their partners are also studied, it is also possible to determine if these partners engage in low levels of risk behavior.

The paper also describes the network location and risk links among members of the sub-network of uninfected participants.

## Materials and methods

Research methods have previously been described (Nikolopoulos et al., 2016, 2017), so we do so only briefly here.

*Setting:* The study took place (6/2013–7/2015) in Athens, Greece, where an HIV outbreak among people who inject drugs (PWID) began in 2011 (Paraskevis et al., 2011, 2013, 2015; Nikolopoulos et al., 2015b).

*Laboratory Methods*: HIV testing used a microparticle anti-HIV-1/2 EIA (AxSYM HIV-1/2 gO, Abbott) confirmed by Western Blot (MP Diagnostics). All HIV+ participants were tested by Limiting Antigen Avidity Assay (LAg; Sedia^TM^ Biosciences Corporation) (Duong et al., 2012, 2015; Nikolopoulos et al., 2017). This test is based on antibody maturation to categorize HIV infection as “recent” or “long-standing.” An Optical Density (ODn) score of 1.5 was used as a cut-off for recent infection, with a median of three ODn values ≤1.5 indicating recent infection. This corresponds to a window period of 130 days (Duong et al., 2015). HIV RNA was quantified for all HIV-positive samples with Artus HI Virus-1 RG RT-PCR (Qiagen). Antibody-negative samples in social networks were tested for viremia (and thus acute infection) in pools of 10.

### Questionnaire

Participants were interviewed using a questionnaire containing items on demographics, sexual and injecting behaviors, drug treatment, and antiretroviral treatment. A main focus of this interview was to collect information on participants' network members and the venues where they interacted with risk network members to enable network and venue recruitment. Participants were asked to name people they had injected or had sex with in the prior 6 months; people who injected or had sex in their presence in the prior 6 months; and people who injected, used drugs, or had sex with people the participants had injected or sex with. They were also asked about places they usually visit to use drugs, to have sex, or to meet new sex partners. We worked with respondents to make a list of sex or drug injecting venues; and staff visited venues to recruit participants for the appropriate arms of the study.

### Recruitment

TRIP used social network tracing and venue recruitment methods to locate those who had recently been infected. These methods have been shown to be able to locate infections downstream, upstream, and sideways across infection chains (Friedman et al., 2014). To be eligible for the study, all participants had to be 18 years or older and able to answer the questionnaire.

Recently-infected participants in TRIP were people who were very likely to have acquired HIV in the past 6 months, and included both original participants who were first enrolled in the project and whose networks were subsequently traced (“seeds”), and their network contacts. Long-term infected individuals were TRIP participants (both seeds and their contacts) who had probably been infected more than 9 months ago. The classification of participants as either recently- or longer-term-infected was based on HIV testing histories, LAg ODn values, and viral load levels.

#### Seeds

More specifically, seeds were newly-HIV-diagnosed PWID referred to the study by the allied ARISTOTLE project (Sypsa et al., 2014, 2017; Hatzakis et al., 2015) or other testing facilities. Seeds with LAg ODn ≤1.5 and no indication of advanced disease were classified as recently infected. Most recently-infected seeds also had documented seroconversion in the prior 6 months. Seeds with LAg ODn >1.5, and without documented seroconversion in the last 6 months, were classified as long-term infected. They were matched to recently infected seeds for age (±5 years), risk group, and gender. Many had tested positive for HIV >3 months before their participation in TRIP but learned about their infection shortly before their TRIP baseline interview.

#### Network tracing

The named network and venue members of recently- and longer-term infected seeds were recruited as follows. We recruited injection and sex partners, and other risk environment contacts, for two steps (i.e., the Step 1 network members recruited directly by the seed, and the Step 2 network members recruited by the Step 1 network members). We tested them for HIV. If they tested positive, we conducted LAg tests and measured HIV viral load. People with recent HIV infection in networks were defined as newly diagnosed individuals with documented testing history of negative serology in the last 6 months and/or LAg ODn ≤1.5, without any indication of advanced disease. Antibody-negative samples were tested for HIV RNA in pools of 10 to identify acute infections. To maximize the number of potential highly infectious people recruited, we also recruited the network members of people with “borderline-recent infection” found in networks. People with borderline-recent infection were defined as newly diagnosed individuals with LAg ODn ≥1.5 but with documented (or reliable, self-reported) history of testing HIV-negative within the last 9 months and/or high viral load (>100,000 copies/ml). For analytic purposes, we included people with borderline-recent infection as part of the recently-infected group in the analyses in this paper.

Based on the logic that infection spreads among members of social networks, and that people often find new sexual and injection partners within their social networks, TRIP did not stop when it encountered an uninfected network member but traced the network for at least one additional step (i.e., at least 2 steps from each seed). When recently or borderline-recently infected participants were located in network tracing, their risk and social contacts were recruited for 2 additional steps. For example, if a network member who was 2 steps away from his/her seed was classified as recently infected, we recruited his/her social network members and then the social network partners of those partners.

#### Incentives

Participants received 10 euros for baseline interviews and 5 euros for each network contact they named who participated in TRIP. As part of HIV testing, we provided participants with standard counseling and appropriate referrals to care. Recently and acutely infected participants received expedited assistance.

### Follow-up

Participants were followed up approximately 6 months later. Those who were uninfected at their first interview were offered the chance to be re-tested for HIV infection (and for recent infection and viral load if infected).

### Informed consent

The project was approved by the Institutional Review Boards of the Hellenic Scientific Society for the Study of AIDS and Sexually Transmitted Diseases in Athens and National Development and Research Institutes (NDRI) in New York. All participants provided written informed consent.

### Analyses

Participants recruited in one of four classifications were included in the present analyses: recently-infected seeds, network/venue members of recently-infected seeds; longer-term-infected seeds (LT seeds); and network/venue members of LT seeds. (In these analyses, people with borderline-recent infections were categorized as recently-infected.) Statistical analyses were conducted with SPSS Statistics 21. **Table 2** compares descriptive statistics for members of the subcomponent of negatives with descriptive statistics for the recently-infected and long-term infected participants to whom they are linked. Although not shown in the table, one-way ANOVAs (for continuous variables) and Chi-square tests of independence (for binary variables) were used to compare these groups on all characteristics presented in the table. These tests produce approximate *p*-values that can only be used as heuristic guides because these three subsets of participants were recruited through chain-referral. As such, the sample violates the assumptions of sampling independence that underlie statistical inference.

*Network Analyses* and visualizations were conducted using Visone 2.16. Calculations of Seidman k-core specify subsets of a component whose members are all linked to k or more members of that same subset. In any given component, there can be only one 2-core. There can be multiple k-cores with k >2; all of their members, by definition, are members of the 2-core. Participants who are not members of a core with k >1 are only weakly tied to the network and thus to patterns of viral transmission. Thus, k-core analysis lets us understand how the uninfected component members “fit into” the large connected component, and the extent to which they are linked to denser parts of the network.

## Results

**Figure 1:**
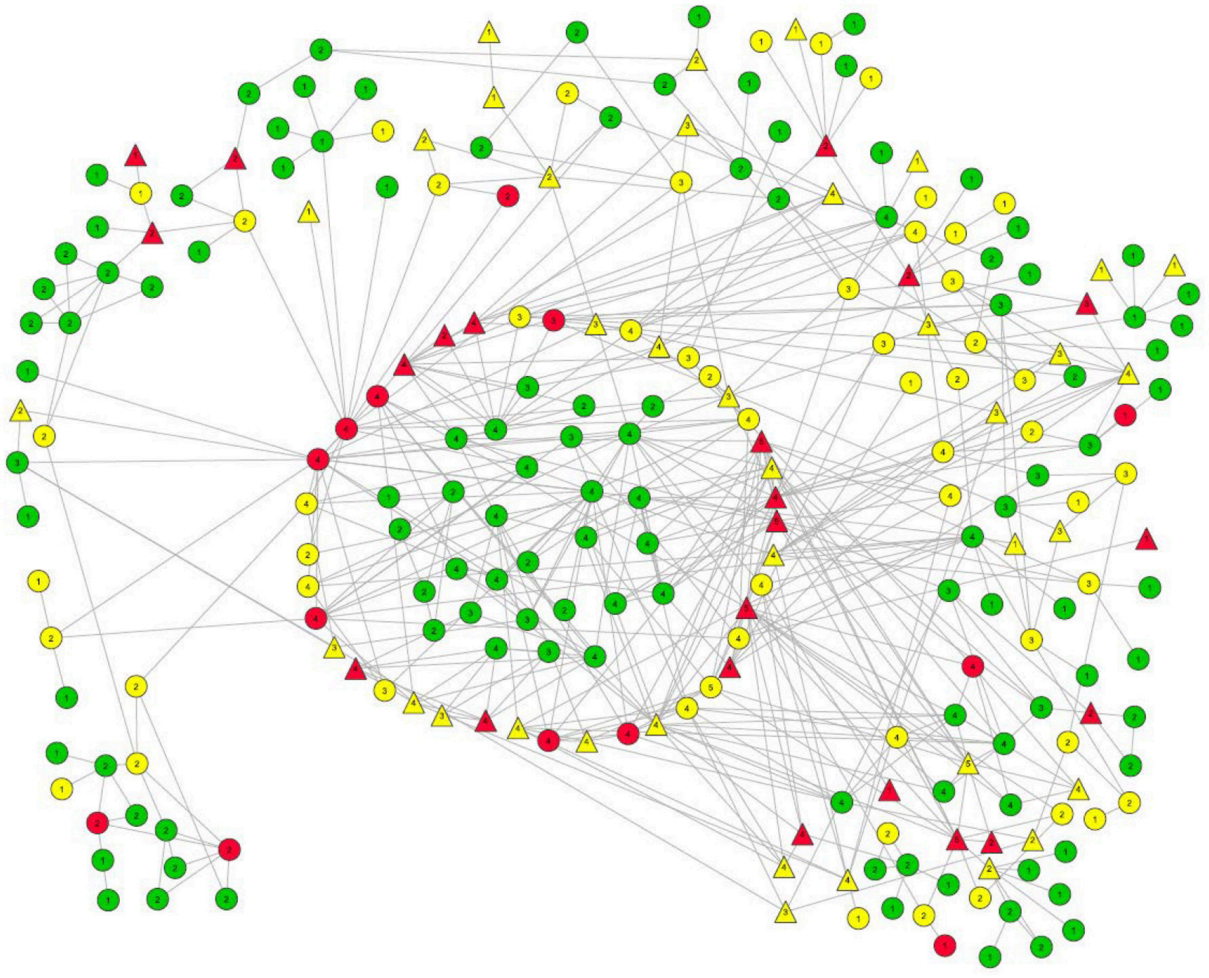
Largest risk network component in the TRIP Athens data. Members of the pocket of non-infection that is the focus of this paper are located in the center. The ring directly around them is comprised of their direct alters. Other members of this large component ate located around the perimeter. **HIV infection status**: Long term positives are marked in yellow.Recently-infected are in red.Uninfected persons are in green.Numbers in the node symbols represent what K-level of the Seidman k-core the participant belongs to. Thus, if there is a “3” inside the node symbol, that participant is linked with at least two other members of the 3-core, and each member of that 3-core is linked to at least two other members of that 3-core. **Viral load**: Triangles represent those with viral load >100,000 copies/mL.Everyone else is represented by a circle. **Link types**: Solid line = Injection LinkDash line = Sex LinkSmall-Dotted Line = Both Sex and Injection Link.

Forty-five recently-infected, 105 long-term infected, and 181 uninfected participants were recruited. The largest connected component had 241 members, and is shown in Figure 1. Within this large connected component there was a subcomponent (i.e., “pocket”) of 29 connected uninfected PWID (located in the center of Figure 1). These 29 participants and the participants with whom they had a direct risk network link are the focus of this paper. (A direct network link usually means that at least one of two participants named the other as a network member during the interview. However, we also considered participants to be directly linked in cases where our field staff saw them together at injection venues and therefore categorized them as people who probably injected together, even if they did not report this on their questionnaires. Only 4 such links were identified among our 29 negative pocket members and any of their direct network connections.)

All but one of the members of this 29-member subcomponent are members of the Seidman 2-core of the large component, as are all of the infected participants to whom they are directly linked. Indeed, most of the 29 are members of a 3-core as well.

**Figure 2:**
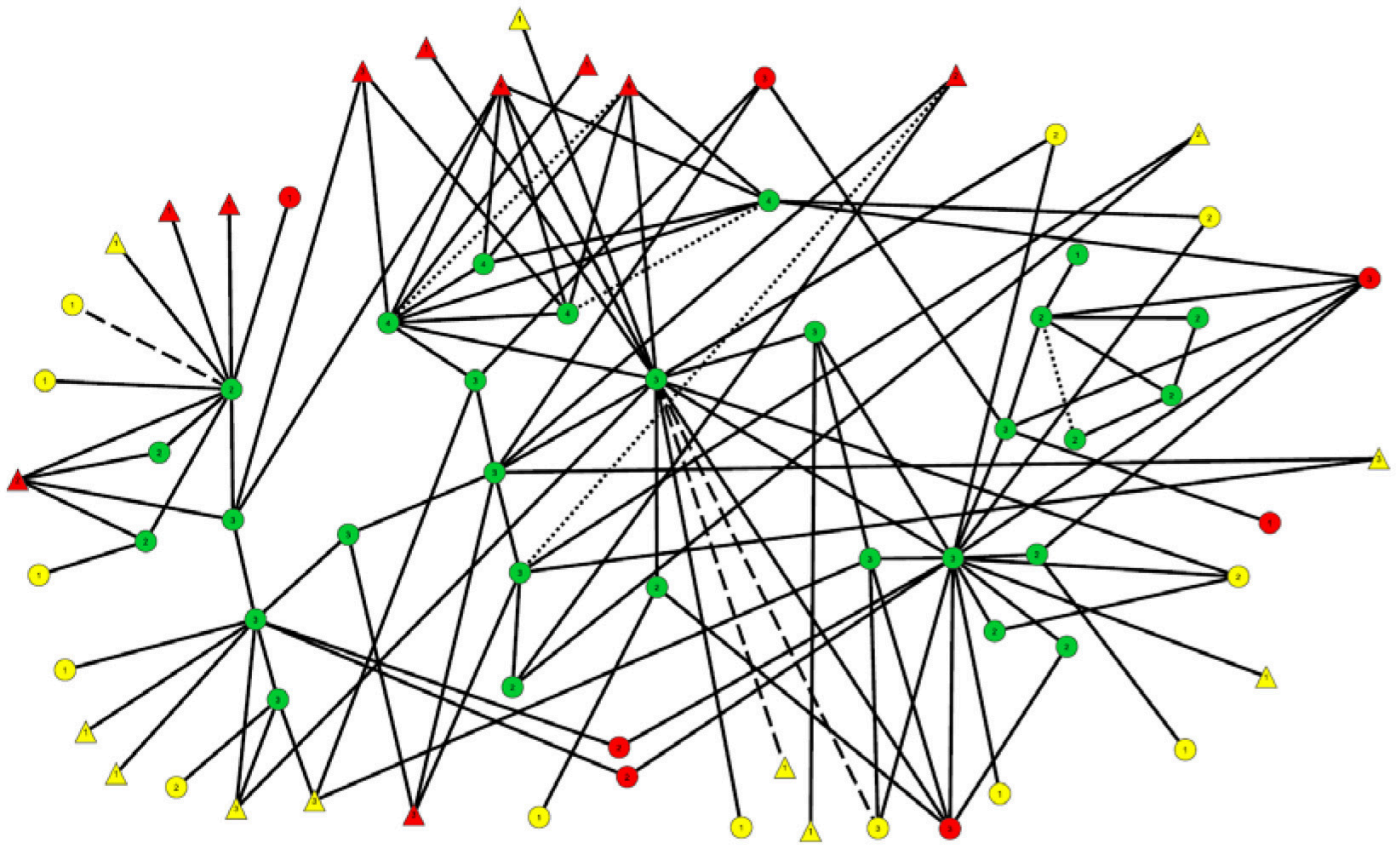
Subnetwork of 29 uninfected participants and the recently- and long-term infected to whom they are linked. The nodes and links in this diagram are the same as those in the center of Figure 1, although their locations have been modified to make this diagram easier to interpret. **HIV infection status**: Long term positives are marked in yellow.Recently-infected are in red.Uninfected persons are in green. **Viral load**: Triangles represent those with viral load >100,000 copies/mL.Everyone else is represented by a circle. **Link types**: Solid line = Injection LinkDash line = Sex LinkSmall-Dotted Line = Both Sex and Injection Link.

Figure 2 shows the 29 members of the connected subcomponent of uninfected participants and their risk ties to each other and to the 17 recently-infected and 24 long-term infected participants with whom they have direct risk-network connections. Table 1 shows that the uninfected had many links with each other (35 total links in Row 1) and with members of the recently-infected (47 total links in Row 2) and longer-term infected (36 total links in Row 3) participants, and that almost all of these risk links were injection links rather than sexual links.

**Table 1:** Numbers and kinds of links of members of the 29 member subcomponent of uninfected participants with each other and with the 17 recently infected participants and long-term infected participants.

	**Number of injection-only network connections**	**Number of sexual-only network connections**	**Number of network connections where both participants both have sex and inject drugs with each other**	**Number of venue-based connections**
Subcomponent of 29 linked HIV-uninfected	33	0	2	0
Those 17 recently-infected participants with whom a subcomponent member has a direct risk network connection	45	0	2	2
Those 24 long-term–infected participants with whom a subcomponent member has a direct risk network connection	33	3	0	2

*For the two venue-based links between uninfected and recently-infected participants, there were no “risky links” since at least one of the dyad members reported not engaging in any sex without a condom and also no syringe sharing. The two venue links in the last row are both cases in which long-term infected participants were recruited from the venues of negatives in the uninfection pocket. In both of these links, each of the dyad members reported syringe sharing, although we do not know if they ever shared with each other*.

Table 2 presents sociodemographic and behavioral characteristics, HIV prevalence rate, and selected other variables for each of these 3 groups and for the total sample. As mentioned above, statistical comparisons only produce approximate *p*-values due to violations of sampling assumptions. Only one comparison was significant at *p* < 0.05: that the long-term infected were more likely to be unemployed.

**Table 2:** Sociodemographic and other characteristics of study participants by major analytic category^*^.

	**Total sample**	**Subcomponent of 29 linked HIV-uninfected**	**Recently-infected participants with whom a subcomponent member has a direct risk network connection**	**Those long-term–infected participants with whom a subcomponent member has a direct risk network connection**	**Total of the 29 member subcomponent of uninfected participants and of the infected participants to whom they are linked**
Total	331	29	17	24	70
Males	260 (78.6%)	26 (89.7%)	13 (76.5%)	17 (70.8%)	56 (80.0%)
Median age in years (IQR)	34 (30–40)	35 (30–39)	35 (32.5–49)	31 (28.25–41)	34 (29–42.5)
Education–at least high school (11 years) completed	129 (40.0%)	9 (31.0%)	6 (35.3%)	10 (41.7%)	25 (35.7%)
Homeless	81 (24.5%)	10 (34.5%)	9 (52.9%)	14 (58.3%)	33 (47.1%)
PWID (injecting over the last 6 months)	304 (91.8%)	29 (100%)	17 (100%)	24 (100%)	70 (100%)
Median duration of injection in years (IQR)	13 (7–18)	10 (5–16)	10.5 (5.75–15.5)	9.5 (4.25–15.75)	10 (5–15.5)
On drug/alcohol treatment at enrollment	124 (37.5%)	5 (17.2%)	3 (17.6%)	6 (25.0%)	14 (20.0%)
Unemployed/unable to work	229 (69.2%)	17 (58.6%)	14 (82.4%)	21 (87.5%)	52 (74.3%)
Sex workers	30 (9.1%)	2 (6.9%)	1 (5.9%)	3 (12.5%)	6 (8.6%)
Male sex workers (% of males)	8 (3.1%)	1 (3.8%)	0 (0.0%)	0 (0.0%)	1 (1.8%)
Female sex workers (% of females)	22 (31.0%)	1 (33.3%)	1 (25.0%)	3 (42.9%)	5 (35.7%)
HIV prevalence rate	45.3%	0%	100%	100%	58.6%
Recently HIV infected	45 (13.6%)	0 (0%)	17 (100%)	0 (0%)	17 (24.3%)
Mean number sex partners (S.D.)	4.3 (17.8)	2.0 (2.5)	3.2 (7.1)	11.4 (41.5)	5.5 (24.6)
Reported condomless sex with at least some partners	160 (48.3%)	11 (37.9%)	10 (58.8%)	5 (20.8%)	26 (37.1%)
Mean proportion of partners with whom participants reported always using condoms (S.D.)	0.46 (0.45)	0.61 (0.44)	0.41 (0.45)	0.72 (0.46)	0.58 (0.45)
Mean number injection partners (S.D.)	10.3 (30.2)	10.3 (14.6)	4.7 (3.1)	12.8 (21.2)	9.8 (15.8)
Reported receptive syringe sharing with at least some partners	112/297 (37.8%)	14 (48.3%)	10 (58.8%)	7 (29.2%)	31 (44.3%)
Reporting giving personally used syringes to at least some partners	107/297 (36.0%)	17 (58.6%)	12 (70.6%)	7 (29.2%)	36 (51.4%)
Median reported categorical frequency of injection	Once per day	2–3 times per day	2–3 times per day	2–3 times per day	2–3 times per day
Mean estimated proportion of injection partners with whom participants engaged in receptive syringe sharing	0.07 (0.15)	0.08 (0.17)	0.12 (0.21)	0.05 (0.10)	0.08 (0.16)
Viral load >100,000 copies/mL	74 (22.4%)	0 (0%)	10 (58.8%)	11 (45.8%)	21 (30.0%)

* *Cell entries take the form of n (%)*.

Twenty-one (72%) of the 29 uninfected “pocket” members were directly linked (network distance = 1) to at least one recently-infected participant, and 16 (55%) to at least one long-term infected participant. We classified 14 out of 29 (48%) uninfected “pocket” members as being at “extremely high risk” because they self-reported syringe sharing and had at least one direct link to at least one injection partner who self-reported sharing syringes and had a viral load > 100,000 copies per mL. These 14 extremely high risk uninfected participants said they used a syringe someone else had already used a mean of approximately 45 times in the last 6 months. Another six of the 29 were linked to someone who shared syringes and had a viral load > 100,000 copies but self-reported that they themselves had not shared syringes in the last 6 months.

Seventeen of the 29 members of the uninfected subcomponent were re-interviewed and had blood taken in a 6-month follow-up. None of these 17 tested positive for HIV. At study intake, 12 (70.6%) of the 17 were in at least one partnership defined as having “extremely high risk.”

## Discussion

The research in this paper shows both the power of risk network research and the limitations of current theories about the spread of HIV (and perhaps other agents) through networks and communities. Unlike phylogenetic research or behavioral epidemiology, the network design used in this study can investigate the ties among people who are infected and uninfected, and thus can pose questions about why groups of people who are uninfected remain that way despite having risk network links to people who both have high viral loads and engage in risky behavior.

Of note, neither of the two existing network-level theories can explain why the 29-member subcomponent remains uninfected. These 29 members have many sexual and/or injection ties both to recently-infected and to longer-term-infected participants, which shows that the networks do not create a bottle-neck that is preventing transmission to the 29 member subcomponent. Similarly, the large number of sexual and/or injection ties to participants who have high viral loads and/or are recently-infected shows that something besides the firewall effect is protecting the subcomponent members.

In a study of HIV in New York in the early 1990s (Friedman et al., 1997), we showed (1) that membership in the 2-core of the large component was associated with being HIV-infected and also with higher levels of risk behavior, and (2) that 3-core membership was also associated with additional risk. Thus, it is particularly puzzling to find a large subcomponent of the non-infected with most of its members in the 2-core (and, indeed, many are in a 3-core), and thus not peripheral to the risk network.

Insofar as we can test them, behavioral theories also do not explain why the uninfected subcomponent remains uninfected. Syringe sharing on the part of both the uninfected and their infected injection partners is widespread, and many of these infected partners have high viral loads and thus should be quite infectious. Nonetheless, since we lack relationship-specific data about how often a given participant engaged in a risk behavior with a specific other participant, it remains possible that the uninfected people whom we designated as “extremely high risk” may not have engaged in syringe sharing with their participant partners who had high viral loads and who also engage in syringe sharing (with unknown persons). A related limitation is that four of the 48 links of uninfected participants with recently-infected participants were “venue links,” which means that we cannot be certain that they are directly linked as friends or partners.

One possible explanation for the fact that the “pocket” members remained uninfected is that the epidemic outbreak among Athens PWID is fairly new-it started in 2011 (Paraskevis et al., 2013; Nikolopoulos et al., 2015a; Sypsa et al., 2017), and that therefore, HIV simply had not reached them yet. We cannot rule this out, but the fact that none of the 17 uninfected participants for whom we have follow-up testing data seroconverted by the 6 month follow-up provides a (low statistical power) piece of evidence that suggests that something more is going on here.

Thus, we are left with a conundrum: none of the existing theories can explain our observations. It is, of course, possible that our data are an anomaly, which suggests that replication research is sorely needed. It is also possible that some members of the pocket of non-infection could have a degree of genetic immunity to HIV (Tsiara et al., 2018). On the other hand, these data are sufficiently strong to suggest that the theoretical development of the field is incomplete and that some deep thinking is required. The focus of this deep thinking should go beyond the question of why individuals with high-risk connections are not infected, and should instead consider the question of how such a large, at-risk connected cluster remains uninfected. Relatedly, if this phenomenon turns out to be common, future efforts should seek to understand the contradiction between this phenomenon and the fact that large-scale epidemic outbreaks do happen.

Future replication research should seek to obtain detailed data on the risk and protective behaviors each member of each dyad engages in with each of their specific network members. It should also collect and analyze specimens for measuring possible individual resistance to infection (e.g., via human leukocyte antigen typing).

Future theory and research should not only seek to understand how such a large “pocket” of uninfected network members can remain so, given the observed risks, but should also seek to explore some additional questions posed by the present findings: (1) Given the large number of longer-term infected participants with viral loads >100,000 copies/mL in Table 2, as these people with extremely high viral load develop more effective antibody responses and their viral loads decrease, will this establish effective firewalls to reduce further viral transmission? And (2) as a corollary question, in the context of an epidemic among Athens PWID that began in 2011 and had just passed its period of highest incidence at the time TRIP began recruiting, why were these high viral loads so prevalent?

Finally, the straightforward methods used here to study subnetworks of non-infection provide a template for studying “bottleneck” and “firewall” network hypotheses in practice. This template should be useful as additional theories are developed.

## Author contributions

All authors contributed to design and data collection, and critiqued and approved the final text. LW had primary responsibility for analyses. LW and SF had primary responsibility for writing the article. SF had primary responsibility for the project as a whole (all sites), while GN took primary responsibility for the conduct of the research at the Athens site.

### Conflict of interest statement

The authors declare that the research was conducted in the absence of any commercial or financial relationships that could be construed as a potential conflict of interest.
